# Supplementary material for: Adolescent Verbal Memory as a Psychosis Endophenotype: A Genome-Wide Association Study in an Ancestrally Diverse Sample
Source: Genes (Basel). 2022 Jan 3;13(1):106. doi: 10.3390/genes13010106 (PMC8774761; doi:10.3390/genes13010106)
Supplement: Supplementary file 1 [file genes-13-00106-s001.zip › Supplementary_materials.pdf]

Supplementary methods

Genotyping and quality control

In the ABCD Study®, the Rutgers University Cell and DNA Repository (RUCDR) genotyped participants with the Affymetrix NIDA SmokeScreen Array, which contained 733,293 SNPs [45]. DNA was extracted using the Chemagen based/Chemagic STAR DNA Saliva4k Kit (CMG-1755-A) and genotyping was done using the Affimetrix GeneTitan Instrument, with steps including DNA fragmentation, labelling, ligation, and hybridization. Samples from either saliva and whole blood were included, whichever had successful call rate calls, higher non-missing rate, matched genetic sex, or less excessive identity by state. RUCDR performed DNA quality controls based on calling signals and variant call rates. The ABCD Data Analysis, Informatics & Resource Center (DAIRC) subsequently performed study-based quality control following the RICOPILI pipeline [46], including 1) removing samples with call rate < 0.9; 2) removing SNPs with call rate < 0.9; 3) removing samples with sex violations. This resulted in a sample of 11,099 participants with 516,598 genetic variants. We removed individuals from one problematic plate (associated with failures in quality control, as advised by the ABCD study), which resulted in a final sample of 11,017 participants.

Relationship inference and principal component analysis

As the ABCD Study® included an ancestrally diverse sample with a combination of singletons, siblings, and twins, it is important to estimate the relatedness and population structure in the sample before conducting the GWAS. We used KING-robust 2.2.5 to infer the unadjusted kinship coefficient for each pair of participants in the sample based on the genotyped data after quality control [54]. The kinship coefficient is defined as the probability that two alleles selected at random from two individuals are identical by descent [54]. The kinship coefficient for monozygotic twins is expected to be 2ˆ(−2/2) = 0.5, and that for first-degree relatives is 2ˆ(−4/2) = 0.25 [54]. Thus, 2ˆ(−3/2) = 0.354 was used as the threshold differentiating monozygotic twins and first-degree relatives, and the same rule applies to second-degree, third-degree, and more distant relationships [54].

We then performed a principal components analysis using the PC-AiR package [38] and the output from KING-robust. This method accounts for sample relatedness. Genotyped data were filtered and pruned using the SNPRelate package in R with a MAF threshold = 0.05, a linkage disequilibrium threshold = √0.1, and a maximum sliding window of 10^6 bp [55]. Any pair of individuals with a kinship coefficient greater than 2ˆ(11/2) = 0.022 (less than fourth-degree relatives) was defined as related, and any pair of individuals with a kinship coefficient less than −0.022 was defined as unrelated. A subset of participants who were ancestrally European was further identified (n = 5,763). Participants with a first principal component < -0.005 and a second principal component < 0.002 were classified as ancestrally European (Figure S2).

To get a more accurate relatedness estimation, we performed a PC-Relate analysis to estimate the familial relatedness adjusted for ancestral background [56]. The unrelated subset of the sample and the first eight (for the overall sample) or four (for the European subset) ancestrally representative PCs estimated in the previous steps were used to estimate the relationships. In this way, PC-Relate generated a kinship matrix with an adjusted kinship coefficient between each pair of individuals. We set any adjusted kinship coefficients < 0.022 to 0 to improve the processing speed in the following GWAS.

Imputation

The ABCD Study® performed imputation using the TOPMed imputation server [50]. Pre-imputation steps were performed according to https://topmedimpute.readthedocs.io/en/latest/prepare-your-data/, including: 1) calculating allele frequencies using PLINK v1.9 [52]; 2) checking .bim files against the Haplotype Reference Consortium and the 1000 Genomes Project for consistencies [47,48], c) converting to VCF files using PLINK v1.9 [52]; and d) running the checkVCF.py to verify that VCF conversion was successful. The VCF files were uploaded to the TOPMed Imputation Server and imputation was performed using mixed ancestry and Eagle v2.4 phasing [49].

Post-imputation

We downloaded the imputed data from the ABCD Data Repository and used bcftools to annotate them with rs IDs based on dbSNP153 [51]. Imputed SNPs with dosage levels were converted to best-guess genotype format using PLINK v2.0 with a hard call threshold of 0.1 [52]. We performed further post-imputation quality control by removing SNPs with imputation quality score (r^2) < 0.3 or MAF < 0.01, for the overall multi-ancestry sample and for the European subset separately. 11,229,083 variants remained for the overall sample and 8,665,039 variants remained for the European subset.

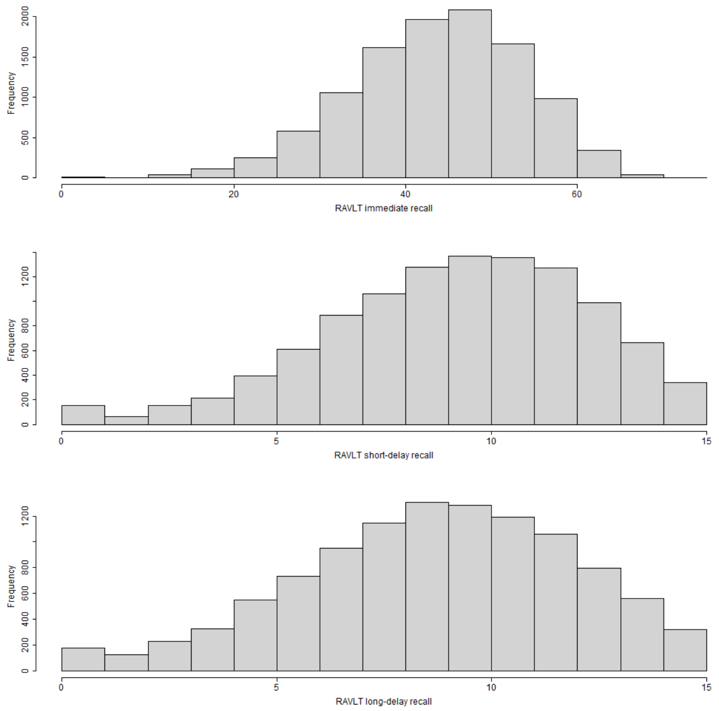

Figure S1. Histograms of the three verbal memory traits. All three verbal memory traits were approximately normally distributed in the sample. RAVLT, Rey Auditory Verbal Learning Test.

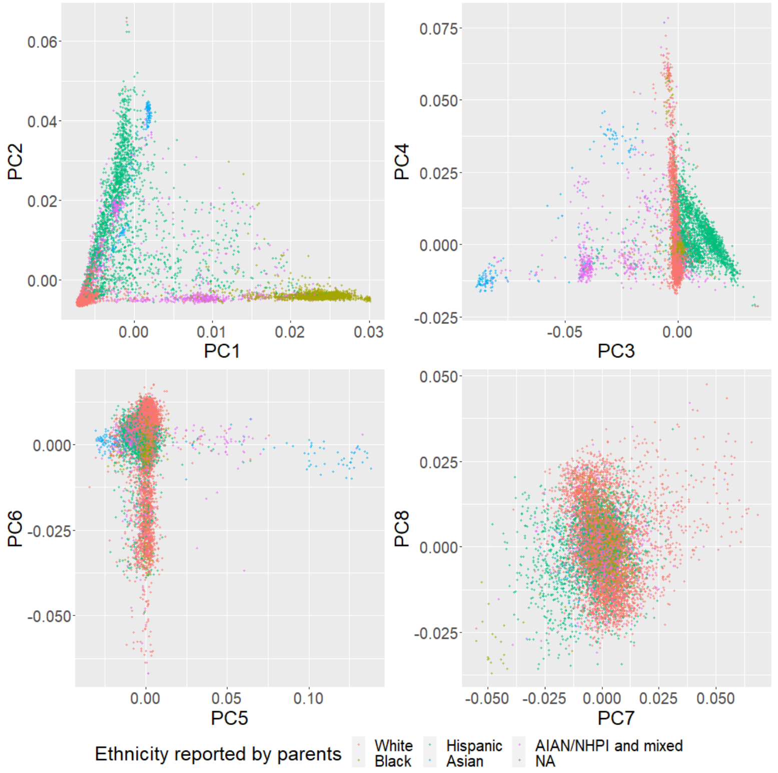

Figure S2. The first eight ancestrally representative principal components coloured by ethnicity reported by parents. NA represents missing data. AIAN, American Indian/Alaska Native. NHPI, Native Hawaiian and other Pacific Islander. PC, principal component.

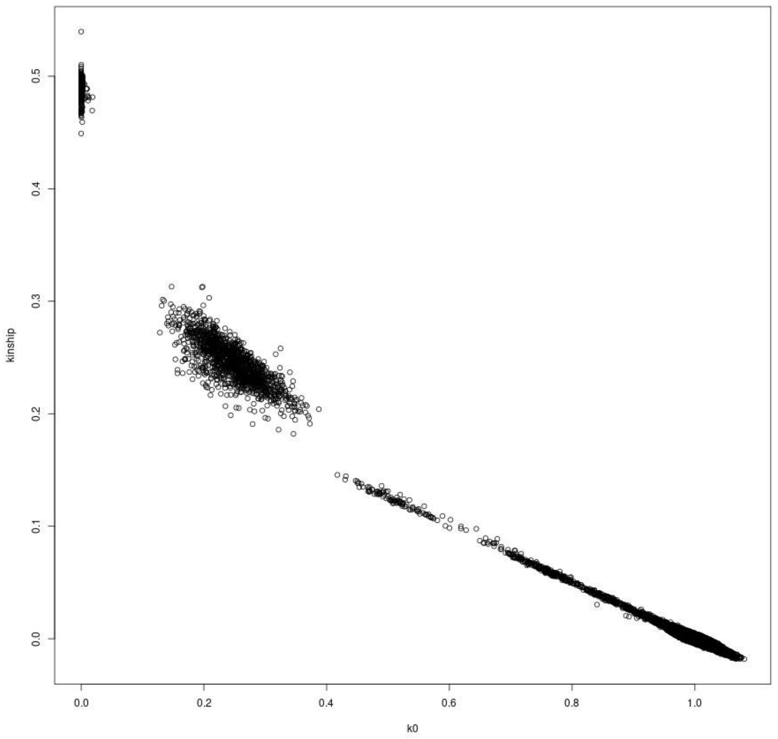

Figure S3. Relatedness estimation adjusted for ancestral background. For each pair of individuals, adjusted kinship coefficients (y-axis) are plotted against k0 (x-axis; the estimated probabilities of sharing zero alleles identical by descent).

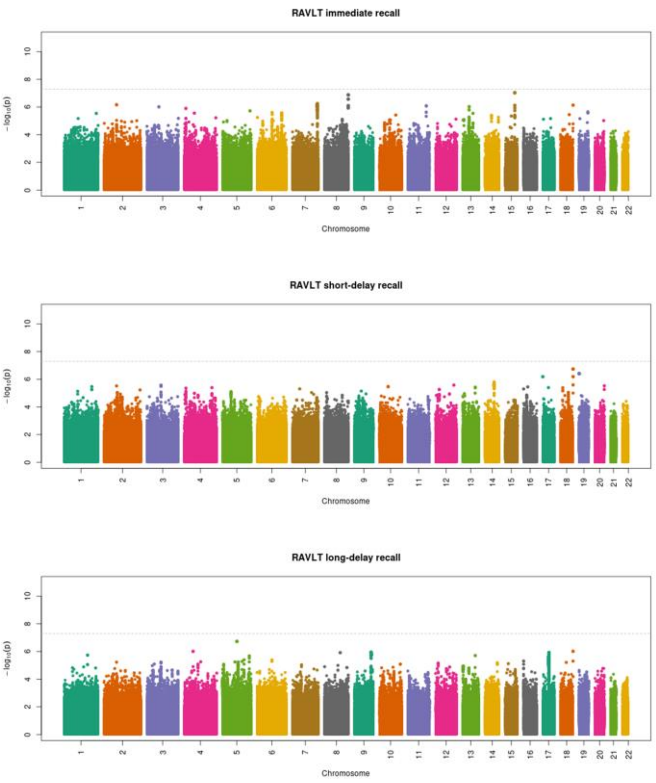

Figure S4. Manhattan plots of the genome-wide association analysis of the three verbal memory traits in the European subset. The dotted line indicates the genome-wide significance threshold of $p = 5 \times 10^{-8}$. RAVLT, Rey Auditory Verbal Learning Test.

Table S1. Results of the genome-wide significant SNPs across the three verbal memory traits

SNP	Chromosome (position)	RAVLT immediate recall		RAVLT short-delay recall		RAVLT long-delay recall	
		Beta (SE)	*p*	Beta (SE)	*p*	Beta (SE)	*p*
rs73984566	2 (191566282)	−1.512 (0.484)	1.80×10^{-3}	**−0.847 (0.151)**	**1.86×10^{-8}**	−0.566 (0.159)	3.55×10^{-4}
rs9896243	17 (46748690)	−0.778 (0.169)	4.39×10^{-6}	−0.194 (0.052)	2.14×10^{-4}	**−0.309 (0.055)**	**2.22×10^{-8}**

Note. Genome-wide significant associations are highlighted in bold. Genomic positions are based on GRCh38. RAVLT, Rey Auditory Verbal Learning Test. SE, standard error.

Table S2. FUMA-mapped genes based on the identified genome-wide significant loci

Lead SNP	Chromosome	Mapped gene	Positional mapping	eQTL mapping	Chromatin interaction mapping
rs73984566	2	NABP1			Fetal_Cortex
rs73984566	2	TMEFF2			Fetal_Cortex
rs9896243	17	CRHR1		Cortex	
rs9896243	17	SPPL2C		Cerebellar_Hemisphere<br>Cerebellum<br>Frontal_Cortex_BA9	
rs9896243	17	MAPT		Caudate_basal_ganglia Cerebellum	
rs9896243	17	KANSL1		Cerebellar_Hemisphere Cerebellum	
rs9896243	17	LRRC37A		Amygdala<br>Anterior_cingulate_cortex_BA24<br>Caudate_basal_ganglia<br>Cerebellar_Hemisphere<br>Cerebellum<br>Cortex<br>Frontal_Cortex_BA9<br>Hippocampus<br>Hypothalamus<br>Nucleus_accumbens_basal_ganglia<br>Putamen_basal_ganglia<br>Spinal_cord_cervical_c-1<br>Substantia_nigra	
rs9896243	17	LRRC37A2		Amygdala<br>Anterior_cingulate_cortex_BA24<br>Caudate_basal_ganglia<br>Cerebellar_Hemisphere<br>Cerebellum<br>Cortex<br>Frontal_Cortex_BA9<br>Hippocampus<br>Hypothalamus<br>Nucleus_accumbens_basal_ganglia<br>Putamen_basal_ganglia<br>Spinal_cord_cervical_c-1<br>Substantia_nigra	
rs9896243	17	ARL17A		Amygdala<br>Anterior_cingulate_cortex_BA24<br>Caudate_basal_ganglia<br>Cerebellar_Hemisphere<br>Cerebellum<br>Cortex<br>Frontal_Cortex_BA9<br>Hippocampus<br>Hypothalamus<br>Nucleus_accumbens_basal_ganglia<br>Putamen_basal_ganglia	
rs9896243	17	NSF	Yes	Cerebellum	

Note. Genes are mapped based on lead SNPs of the loci and SNPs in the same linkage disequilibrium ($r^2 \geq 0.6$).
